# Botulinum Toxin: Previous Developments, Current Status, and Perspectives

**DOI:** 10.3390/toxins17100480

**Published:** 2025-09-26

**Authors:** Wolfgang H. Jost, Alexandre Kreisler

**Affiliations:** 1 Parkinson-Klinik Ortenau, 77709 Wolfach, Germany; 2 Department of Neurology, University of Saarland, 66421 Homburg, Germany; 3 Department of Neurology and Movement Disorders, CHU Lille, F-59037 Lille, France

## 1. Introduction

For many years, botulinum toxin has been successfully applied in various fields. While other indications for botulinum toxin soon emerged, such as rehabilitation, dermatology and aesthetics, urology, and pain therapy, the majority of its applications are in the field of neurology [1]. In the field of rehabilitation, it is generally used the most frequently and over long periods of time, with a focus on spasticity, followed by aesthetics. Due to its many potential indications, botulinum toxin is also being adopted in many specialist areas. Initially, this led to considerable uptake, but this has now slowed down somewhat, opening the way for new manufacturing developments among manufacturers subject to various influences (of which the expected sales play the biggest role). While small groups of motivated users previously showed interest in discovering the potential applications of botulinum neurotoxin (BoNT) and engaged in extensive collaborations with each other, nowadays, BoNT has a very diverse group of users who generally only apply it in cases of innovative individual indications, i.e., primarily doctors who use it therapeutically. This point is illustrated well in this Special Issue by two articles devoted to truncal dystonia and freezing of gait [2,3]. As a result, the early innovative spirit of initial BoNT usage has declined, and the number of its scientifically active users has significantly decreased. New publications on the topic are generally limited to reviews containing few original papers, and new ideas and approaches are scarce. As a result, the number of BoNT users will decrease, and existing patients may either no longer receive care in some areas or have difficulty finding a practitioner. Several questions arise: Do we want to accept this, or are new initiatives desirable? How can we positively influence these developments? Above all, how do we ensure that our patients can receive long-term treatment?

The use of BoNT in medicine is an incredible success story. One could say that previously, traditional medical practices were in accord with the modern more scientific approach. In simple terms, people would observe something, then investigate their observations and apply their results to other questions. This was less a pure scientific approach and more resembled the approach of empirical medicine. Today, we are more likely to begin with a question and scientifically investigate how best to solve it. Of course, the current approach is an improvement, particularly in terms of productivity, but we should not neglect clinical observations; rather, we should determine whether they would benefit from further investigations. As an example, many practitioners began using botulinum toxin in the 1980s because they wanted to temporarily paralyze individual muscles without causing permanent damage. Subsequently, the therapeutic uses of BoNT were explored in more detail both clinically and scientifically, but some findings emerged more by chance and, in the further course of events, proved just as important as the specific questions posed by preliminary scientific studies. Both approaches led to progress and could explain the success story of therapeutic BoNT usage.

## 2. Current Problems

In the early years of BoNT exploration, two approaches were prevalent: clinical experience and everyday observations on the one hand, and disciplined scientific work on the other. Working groups were small and thus, even on the international level, still manageable, which led to intensive exchanges. Workers in the field not only read published papers, but also often discussed them with the authors. Both sides influenced each other and were able to provide input, and thus also prevent undesirable developments.

This led to some difficulty in translating scientific ideas into practice, and subsequently to further difficulty in successfully investigating clinical observations using reliable studies. Today, BoNT has been approved for use in cases where efficacy has been demonstrated, but the fundamentals are unfortunately not much better understood than before its use.

We also have cases of BoNT approval where, in retrospect, the relevant studies demonstrated significant deficiencies or did not truly answer the relevant questions [2]. However, once an approval has been granted, the sponsor is no longer particularly concerned because, on the one hand, they have no desire to jeopardize their approval, and, on the other, they reject the idea of investing a great deal of effort without the likelihood of financial benefit. Industry-sponsored studies to explore older indications for BoNT exist [4] but are rare; in actuality, manufacturers are somewhat timid and see no benefits in such investments. When performing such studies, practitioners face other pitfalls such as a lack of financial resources and personnel. The current Special Issue, however, presents two excellent studies on BoNT usage for treating hemifacial spasm and cervical dystonia, which have previously seen limited exploration [5,6]. As an example of our point above, we take the approval of BoNT for treating migraine. The study in question was a veritable breakthrough [7], but unfortunately, simply because approval had been granted, no follow-up studies of the same quality were conducted. It is clear that our objectives are not the same. We may well be partners, but as members of the medical profession we are in no way mere helpmates for industry: we are responsible for our patients first and foremost.

As another case in point, we take the most significant area of BoNT use: spasticity. In the original approach, the toxin’s effect on spasticity was easily explained because it was assumed that the initial goal was only to reduce muscle tone. However, it became clear over time that, on the one hand, this benefit is difficult to demonstrate in some cases, and, on the other hand, new findings could even jeopardize these previous achievements. However, to gain approval, a good idea is not sufficient; financial support from manufacturers is also required. Accordingly, study design is undeniably driven by the desire for approval and by the conditions of manufacturers and agencies, rather than by clinical needs and ideas. The influence of these authorities, in particular, strongly influences approval, so compromises are often sought and the actual issue is only partially addressed. Therefore, from the start, symptoms and complaints must be taken as the primary factors for study, rather than the possibility of receiving approval and how best to achieve it. Studies must not be understood as being influenced to any degree whatsoever by the possible objectives and reservations of the approving agency.

## 3. Problems with Current Studies

This also creates problems with current studies. While all ideas and even mistakes were previously accepted, studies are now much more complex and are only funded if they can promise success, especially economic success. This often means that only a limited “core group” is covered, as this is easier to define and carries less risk of obtaining a final non-significant result. As a result, many clinically relevant questions remain unanswered: Where can I obtain clear study results with reasonable effort? When are the dosages used strong enough to make the high effort worthwhile? “Minor” problems and questions are not being answered at all, as they will either be unlikely to lead to approval; the approval would not have significant clinical or financial relevance; or the improvements in findings would be too difficult to measure. We are therefore losing the chance to study therapeutic effects because manufacturers are more interested in costs and revenues, regulatory agencies are not prioritizing therapeutic effects and patient benefits, and we do not want to do pure art for art’s sake. The current studies in the field therefore only partially meet patient needs, as the primary goal is not the question itself, but rather the financial considerations of manufacturers and payers.

Consider a further example: the possible use of BoNT to treat striatal toe via injection into the extensor hallucis longus muscle [8]. This positive effect has been known for a long time, but no approval studies have been conducted because

1. The results would be of little clinical and scientific significance.
2. The financial benefit for the manufacturer would be low, even compared to the effort involved, since only a small amount of BoNT would be injected and the final proof of benefit would not be easy to define; alternatively, it would be difficult to convince agencies of the real benefits. In comparison, in the treatment of leg spasticity, there are relatively “simple” parameters, relatively high dosages, and evidence of effectiveness that is easy to communicate even to laypeople.

In summary, one can say that botulinum toxin therapy was initially a truly unimaginable success story. It did not involve seeking the best therapy for a disease, but rather seeking potential areas of application and therapy. In retrospect, one could argue that many unnecessary attempts were also made, but this also offered the opportunity for learning about previously unknown options. For example, I (WHJ) used botulinum toxin to treat an anal fissure and achieved good results [9]. This therapy has only achieved limited adoption, partly due to its financial and administrative costs, but it is also currently receiving renewed attention.

A therapeutic gap in dystonia and spasticity has been closed, but has remained limited to approved indications. While it was initially possible to decide on new indications and even participate in approval trials, this became increasingly rare over time. In the second wave of BoNT studies, therapy was optimized by, for example, improved anatomical knowledge and improving injection accuracy using ultrasound [10]. However, these further developments were often left in the hands of experienced practitioners, so that their younger colleagues, while interested, rarely played a leading role (with the exception of ultrasound, where younger colleagues had a major influence on technique development and to an extent still do). So, what is next? If we fail to interest our younger, ambitious colleagues, no new indications for BoNT usage will be discovered, and existing applications will not be optimized. This would be a great loss, since, in our opinion, the BoNT success story is far from over, and we have not yet investigated many well-known options and not even considered some potential ones, such as the various non-motor effects of BoNT [11]. We need to continue to work on this topic intensively so that we do not neglect therapeutic options. Sometimes it helps to talk about it, discuss it, and, above all, reflect on it. With this Special Issue, we hope to make a contribution by collecting new ideas and approaches, exchanging our different experiences, and overcoming deficits. This research is mainly academic and is not essentially concerned with approvals or profits, but rather with developing the subject matter.
